# Telephone cognitive behavioural therapy to prevent the development of chronic widespread pain: a qualitative study of patient perspectives and treatment acceptability

**DOI:** 10.1186/s12891-019-2584-2

**Published:** 2019-05-10

**Authors:** Claire Fraser, Marcus Beasley, Gary Macfarlane, Karina Lovell

**Affiliations:** 1 School of Health Sciences, Division of Nursing, Midwifery and Social Work, University of Manchester, Oxford Road, Manchester, M13 9PL UK; 2 Medical Sciences and Nutrition, Health Sciences Building (1st floor), School of Medicine, Foresterhill, Aberdeen, AB25 2ZD Scotland; 3 Greater Manchester Mental Health NHS Foundation Trust, Manchester, M25 3BL UK

**Keywords:** Chronic widespread pain, Telephone cognitive behavioural therapy, Treatment acceptability, Patient perspectives, Prevention, Qualitative

## Abstract

**Background:**

Telephone cognitive behavioural therapy (tCBT) is an acceptable and effective treatment for patients with chronic widespread pain (CWP). Preventing the onset of CWP offers considerable benefits to the individual and society and the MAmMOTH study is the first aimed at CWP prevention. The study is a two-arm randomised trial testing a course of tCBT against usual care for prevention of CWP. This nested qualitative study explores patients’ treatment experiences, with a view to understanding their potential influences on acceptability of the intervention.

**Methods:**

The MAmMOTH Study recruited 1002 participants, half of whom were randomised to receive tCBT. Participants were eligible for invitation to the trial if they had pain for which they had consulted their GP, or had pain and visited a doctor frequently, and had 2 of 3 risk factors for development of CWP. Participants randomised to tCBT who had completed treatment were eligible for invitation to qualitative interviews for this study. Individual qualitative interviews were conducted with a sub-sample (*n* = 33) of patients at high risk of developing CWP who had been allocated to the intervention arm. Semi-structured telephone interviews explored treatment experiences and intervention acceptability. Data was analysed using Framework analysis.

**Results:**

Participants presented with a range of musculoskeletal and auto-immune conditions and almost half described their pain as ‘chronic’ on study entry. Many participants perceived the trial intervention to be aimed at treatment of pain rather than prevention of pain. Initial expectations prior to treatment varied, with scepticism more likely for those who had little prior knowledge of CBT approaches. All participants provided positive feedback post intervention particularly in relation to the modality, therapist experience and skills and the intervention. The majority of participants described positive changes in either their subjective level of pain or pain-management post-intervention and some attributed the positive change directly to the intervention as a result of empowerment, increased self-management and cognitive restructuring.

**Conclusions:**

This study extends our understanding of the acceptability and suitability of preventative interventions for chronic widespread pain and provides further evidence for the acceptability of tCBT.

**Trial registration:**

Clinical Trials.gov NCT02668003 (registered 29th January, 2016).

**Electronic supplementary material:**

The online version of this article (10.1186/s12891-019-2584-2) contains supplementary material, which is available to authorized users.

## Background

There is increasing evidence that psychological therapy delivered by telephone is accessible and effective for a range of conditions and it offers particular advantages by allowing those with disabilities, childcare and employment commitments and geographically remote patients to access health services without the need for scheduled face-to-face appointments [1, 2].

With regard to cognitive behavioural therapy (CBT) in particular, evidence suggests that CBT delivered by telephone is superior to ‘treatment as usual’ for a number of conditions [3], and the recent MUSICIAN study has demonstrated treatment efficacy, cost effectiveness and acceptability for patients with Chronic Widespread Pain (CWP) [4–6].

CWP has been defined as pain lasting longer than 3 months which affects both sides of the body, above and below the waist; it is often associated with fatigue and psychological distress [7, 8]. CWP affects between 11 and 16% of the population [9] and results in considerable costs to the individual and society [10]. Usual care for CWP is based on treatment guidelines which recommend pharmacological, physical and psychological interventions [11]. A multidisciplinary approach is often taken incorporating education about the condition alongside graded exercise, pacing activities, drug therapy, psychotherapeutic interventions and sleep management [12]. Evidence suggests that early intervention and improved prognostic indicators for musculoskeletal pain might be particularly effective since longer duration of symptoms (> 6 months) is linked to poorer outcomes [13, 14].

Moving beyond management to prevention of CWP is thus clearly desirable and now possible due to the availability of prediction models which can identify those at high risk of developing CWP. The Maintaining Musculoskeletal Health (MAmMOTH) study [15] is testing the hypothesis that among patients who are identified as at high-risk, a short course of telephone-delivered CBT (tCBT) can prevent the development of CWP when compared to usual care. This is the first randomised controlled trial (RCT) aimed at CWP prevention.

Successfully implementing research into NHS practice requires that new interventions are accepted and welcomed by those receiving the service. The acceptability of an intervention involves making a normative judgement based on the extent to which it can be tolerated and considered reasonable [9]; acceptability is likely to be a multi-faceted construct involving cognitive and emotional responses [16]. Qualitative methods are therefore the most appropriate method to explore these views as they offer the opportunity to examine what really matters to patients and what might facilitate and hinder an intervention [17] and have been used successfully in other studies exploring the management of CWP [6].

This paper reports the findings of a qualitative study nested within the MAmMOTH RCT, conducted with a sub-sample of participants in the intervention arm who received tCBT. The study aimed to explore participants’ treatment experiences with a view to understanding their potential influences on intervention acceptability. A behavioural approach to the management of chronic pain is well established as being effective and cost effective and we have moved to using this approach in persons who have symptoms which put them at high risk. Whether this particular trial is effective or cost effective we would argue that a behavioural approach to the symptoms marking people as at high risk is very relevant and thus establishing the acceptability is important. In the event that this approach is not (cost) effective understanding issues of acceptability will be relevant for future studies and service delivery. Indeed we think there are some advantages in evaluating the acceptability of an intervention before the main trial results are known – in that interpretation is not influenced by this knowledge. Therefore, the acceptability study has been intentionally conducted and analysed before trial outcomes are known to avoid biased interpretation [18].

## Methods

Potential participants for the MAmMOTH RCT were identified via primary care screening questionnaires distributed to persons registered at selected GP practices within three Scottish Health boards. Patients were eligible if they were identified as being at high risk of developing CWP, determined by the presence of pain for which they had consulted their GP in the previous 6 months, along with 2 out of 3 of the following indicators: high illness behaviour score; high number of somatic symptoms; high sleep problem score.

The telephone CBT intervention (tCBT), delivered to those patients randomised to the intervention arm, consisted of an initial assessment session (45–60 min), 6 weekly sessions (each 30–45 min) delivered over a six week period and follow up ‘booster’ sessions at 3 and 6 months. The sessions involved education (e.g., about musculoskeletal pain and somatic symptoms) and specific CBT techniques (e.g., behavioural activation, activity pacing and identifying and challenging negative and unhelpful thinking patterns). Participants were also supported via a self-management CBT manual [19] to facilitate collaborative goal setting between therapist and patient, diary keeping and exercises to complete after specific sessions. Full details of the trial can be found in the published protocol [15].

The intervention was delivered by BABCP (British Association for Behaviour and Cognitive Psychotherapies) accredited CBT therapists with at least two years’ experience of delivering CBT. All therapists attended a two-day training course on the intervention prior to treatment delivery and received two-weekly supervision from trained and experienced CBT therapists.

Participants were eligible for invitation to the qualitative study if they had completed at least one treatment session and had not been withdrawn from treatment. Participation in the nested qualitative study was voluntary and not a pre-requisite for trial participation. Invitations were sent by letter 12 months post treatment start by the study coordinator to 101 participants. The rationale for inviting 101 was to ensure a sample of around 30 participants could be achieved as this was considered necessary to achieve data saturation [20, 21]. A 30% response rate was considered to be a reasonable estimate to a follow up postal invitation based on the trial team’s experience and other published data [22]. Sampling was purposeful in that we targeted participants who had experience of the trial intervention. Variation in geographical location was achieved by sampling across all three trial recruitment sites. All invited participants who expressed an interest in the qualitative study were requested to return a consent to contact form (*n* = 39); on receipt participants were telephoned to provide further information about the study and to discuss participation. The MAmMOTH trial adheres to CONSORT guidelines and in line with such reporting the participant flow from the trial to this nested study is included in the Participant Flow diagram (see Additional file 1).

Semi-structured in-depth telephone interviews were conducted to determine participants’ perspectives on treatment experiences and acceptability. The interview schedule (see Additional file 2), was drafted by CF based on previous qualitative work by members of the team [10] and reviewed by all authors and a patient representative prior to ethics submission. The schedule was organised around three broad topics: context (extent of current difficulties; reasons for participating in the trial); process (expectations and experiences of receiving the intervention; reflections on mode of treatment delivery); outcome (perceived impact of intervention and treatment acceptability). Interviews were conducted by telephone on a pre-arranged date and time (between December 2017 and March 2018), consistent with the treatment delivery approach. All interviewees took part in one telephone interview conducted at least 12 months after the start of treatment. The rationale for this was we wanted to complete collection of the main outcome *before* doing the interviews. If we had done interviews before collecting the 12-month outcome we would be measuring the effect of having the intervention and the interview – it’s possible an opportunity to give feedback about the intervention could change the way participants answered the questionnaire. It’s also possible that participants would be less likely to provide the main outcome if they had just been invited for interview because of ‘research fatigue’. All interviews were conducted by an experienced qualitative researcher (CF) who was independent from the main trial study team and not previously known to participants. Interviews were digitally recorded with the consent of the interviewee and transcribed verbatim. Interview duration ranged from 15 to 57 min; mean (SD) 25.4 (9.1) minutes. Demographic and intervention sessional data was provided by the study coordinator after the interviews were completed.

Data analysis was completed using Framework analysis [23]. Transcripts were read independently by CF and, to enhance the rigour of the study, another member of the research team (KL) to achieve familiarisation with the data and to check data saturation had been reached in line with the study’s aims and objectives. This combined reading of the transcripts by two researchers also facilitated discussion of the emerging themes which were then used to develop a theoretical framework.

Each transcript was then then analysed in detail by CF by indexing data into the theoretical framework in Microsoft Excel using a matrix approach with participants represented as rows and themes represented as columns. During the indexing and subsequent charting process the theoretical framework was revised and refined, for example to capture any newly emerging themes and to merge overlapping themes. The final version of the theoretical framework was agreed by all authors and is supplied as an additional file to this manuscript (see Additional file 3). After consensus on the theoretical framework was reached, the first author re-read all the transcripts to check ‘fit’ with the framework and drafted the paper. All authors commented on drafts of the findings to produce the final manuscript.

## Results

Of the thirty-nine patients returning consent to contact forms, thirty-three went on to participate in telephone interviews. Two patients declined to participate after being contacted by the researcher due to lack of time, two did not respond to attempts to contact and two were not contactable due to incorrect or incomplete contact details provided.

All thirty-three participants had completed the intervention initial assessment and all but one had gone on to receive tCBT treatment and follow up sessions. One participant who had only completed the initial assessment was invited to the acceptability study in error and this participant’s data is not included in the analysis presented in this paper. For the thirty-two patients who went on to access tCBT, the number of treatment sessions completed ranged from 4 to 6; the number of follow up sessions completed ranged from 1 to 2.

The nested sample was largely representative of the trial population in respect of age and gender; interview participants scored slightly poorer on all baseline measures of health (Table 1).

**Table 1:** Baseline characteristics for the trial sample and nested qualitative study

	Total Trial sample (*n* = 1002)		
	Trial participants not interviewed (*n* = 970)	Nested qualitative study participants (*n* = 32)	Difference (95% CI)
Age, years, mean (SD)	57.7 (14.4)	61.4 (12.8)	3.8 (−1.3–8.8)
Female, n (%)	570 (58.8)	19 (59.4)	0.6% (−1.7–1.8%)
Illness Behaviour, mean (SD)	9.8 (3.5)	10.3 (3.2)	0.5 (−0.8–1.7)
Somatic symptoms, any, n (%)	451 (46.5)	16 (50.0)	3.5% (−1.4–2.1%)
Sleep problems score, mean (SD)	10.2 (4.6)	10.2 (4.5)	0.0 (−1.6–1.7)
EQ 5D, mean (SD)	0.70 (0.19)	0.67 (0.11)	−0.03 (− 0.09–0.04)
ICECAP, mean (SD)	0.85 (0.15)	0.83 (0.13)	−0.02 (− 0.08–0.03)
GHQ, > = 2, n (%)	431 (44.9)	15 (46.9)	2.0% (− 15.6–20.0%)
Polysymptomatic distress, mean (SD)	7.1 (3.5)	8.2 (2.8)	1.0 (− 0.2–2.3)

*CI* confidence interval, *SD* standard deviation, *IQR* inter-quartile range

Three main themes emerged from the data: i) Presenting issues and pain management, i.e., the type of pain experienced and help received prior to the trial; ii) Expectations and reflections, which describes trial perceptions and participants’ motivation to participate and reflections on receiving the intervention and iii) intervention impact and acceptability, referring to the perceived impact of the intervention and acceptability.

### Presenting issues and pain management

The first theme, entitled presenting issues and pain management describes the issues which participants presented with at the start of the trial including the nature of pain experienced, pain management strategies employed and help received prior to participating in the trial.

#### Nature of the pain experienced

As might be expected given the focus of the trial, interview participants experienced a range of issues related to musculoskeletal as well as auto-immune conditions as detailed in Table 2. Almost half of those interviewed described their level of pain on entering the study as ‘chronic’ or ‘debilitating’ resulting in significant disruption to their daily lives, particularly in relation to sleep, mobility, mental health and general well-being:

*“I’d been having a lot of pain … it was debilitating and exhausting and because I’m a very busy person, it stopped me from doing things that I basically love doing …*.” *(IV3).*

**Table 2:** Sample Demographics

Interview sample demographics (n = 32)			
Unique identifier	Age at screening	Gender	Related condition
IV1	51	F	Lower back pain, neck pain and migraines
IV2	67	M	Back and leg pain
IV3	77	F	Osteoarthritis
IV4	54	M	Sciatic pain (trapped nerve)
IV5	64	M	Musculoskeletal injuries (shoulder, knee, hip)
IV6	74	F	Musculoskeletal injury (knee) and frozen shoulder
IV7	49	F	Patella femoral joint syndrome
IV8	74	F	Knee pain
IV9	79	F	Osteoarthritis
IV11	47	F	Musculoskeletal injury (back)
IV12	48	M	Musculoskeletal injury (knees)
IV13	67	F	Rheumatoid Arthritis
IV14	50	M	Musculoskeletal injury (shoulder)
IV15	68	F	Ankylosing spondylitis
IV16	63	M	Osteoarthritis
IV17	56	F	Multiple sclerosis
IV18	69	M	Osteoarthritis
IV19	53	M	Sinus pain and migraines
IV20	64	M	Arthritis (knees)
IV21	86	F	Arthritis (knees and hands)
IV22	47	F	Back pain
IV23	41	F	Musculoskeletal injury (back, neck and shoulder)
IV24	48	F	Neck and shoulder pain
IV25	35	F	Migraines and tendonitis (foot)
IV26	62	M	Sciatica (slipped disc and trapped nerve)
IV27	46	M	Inflammatory bowel disease
IV28	72	M	Muscle cramps/leg pain (Parkinson’s)
IV29	70	F	Osteoporosis and Urinary Tract Infection
IV30	64	F	Sciatica
IV31	54	F	Inflammatory pain (Lupus)
IV32	78	M	Knee pain
IV33	75	F	Osteoarthritis

#### Pain management strategies employed

A small number of participants had already adopted their own discomfort and pain self-management strategies, for example, positive thinking, exercising, pacing or resting prior to involvement in the trial and these were used by them to minimise the impact of the pain experienced:

*“Well, I was taking exercise, going out for a walk, and it seemed to help. I stopped taking the tablets before the course started and it was just positive thinking, ignoring the pain, and it was fine …*.” *(IV2).*

#### Help received prior to participating in the trial

The participants interviewed had experienced a wide range of medical and physical interventions prior to trial recruitment. More than half of those interviewed were taking regular medication including analgesics and anti-inflammatory drugs and about a quarter had been referred by their GP for further consultations and/or treatment (including steroid injections and operative treatments). Two thirds of the sample had received manual physical therapy, the majority via NHS physiotherapy services, whilst some had accessed a range of private alternative treatments including osteopathy, hypnotherapy, acupuncture and chiropractic treatments. Other pain management strategies employed included exercise, cryotherapy and heat treatment.

### Expectations and reflections

The second theme to emerge from the data, expectations and reflections, comprised of three main sub-themes: Trial perceptions and motivation to participate, initial expectations prior to therapy and reflections on receiving the intervention.

#### Trial perceptions and motivation to participate

Although this was a prevention trial, targeting participants *at risk of developing* CWP, it seemed that for some participants, the MAmMOTH study was perceived to be a trial focused on managing (rather than preventing) CWP. Although many were motivated to take part as they wanted to support health research, some were surprised to meet the inclusion criteria as they only experienced ‘discomfort’ or ‘irritating’ or ‘not bad pain’:

“*… I was surprised I was even chosen for this study, to be honest … I didn’t think my pain level would have justified being part of this study, because I had such small niggling pains...” (IV7).*

However, for those participants who described experiencing more ‘chronic’ pain, the trial was a welcome opportunity to try a new way of working with their pain.

Some patients appeared to have assessed the potential for gain versus risk when considering whether to participate in the study and judged it to be a non-invasive, low risk, ‘nothing to lose’ opportunity and an alternative to medication:

*“I didn’t think I’d anything to lose. It wasn’t going to make my condition worse. You know, it wasn’t like I was trying a new exercise or a physical exercise that would make the pain worse, so I thought, well let’s just go for this.” (IV22).*

Others highlighted the contrast between the trial intervention and usual GP care, highlighting that taking part was an opportunity to be listened to, to be given time to talk:

*“I feel that I can probably get more from [the trial] talking about this, than I can, like I could a doctor, because he doesn’t have the time … you are very conscious of how stretched they are these days I think. You are. I mean all you have to do is look in the waiting room, don’t you?” (IV6).*

#### Initial expectations prior to therapy

Prior to the first session about a third of participants had fairly low expectations about what the intervention could achieve for them, either due to scepticism about how talking could impact on a physical symptom or because they had doubts about the relevance of this approach for their particular condition:

*“I was very sceptical about it initially because I thought, how can this help me?...the way we were trained as nurses, that we must observe the patient, observe people face to face … I never thought that I could ever respond to a phone call … If you can’t see somebody, how could this work?” (IV3).*

Some participants described themselves as ‘open-minded’ and ‘willing to give it a try’; whilst others said they really didn’t know what to expect but were curious enough to engage with the intervention.

About a fifth of participants had a good understanding of cognitive behavioural approaches and began the trial with high expectations about the benefits of talking therapy in relation to pain management, either based on prior experience of CBT (for other health conditions) or from their own reading or professional roles.

#### Reflections on receiving the intervention

When participants were asked to reflect on the intervention the majority were very positive and their feedback highlights key factors in relation to intervention modality, the therapists delivering the intervention and the accompanying intervention manual.

Commenting on the intervention modality, more than two thirds were completely satisfied with receiving this type of intervention by telephone and saw no additional benefits to be gained by receiving the intervention face-to-face. Indeed many were keen to highlight a range of benefits to be achieved via this modality, for example, confidentiality, anonymity and reduced stigma.

The telephone-based intervention also increased accessibility for those who were working and/or had childcare commitments and those who were geographically remote from NHS clinic sites.

Some participants had mixed views on modality: whilst recognising some of the benefits (as highlighted above) that telephone interventions can offer, they wondered whether face-to-face support would achieve a more personal and holistic approach and enhance the therapeutic process, for example, by incorporating non-verbal communication. Two participants felt the telephone did not afford a sufficiently in-depth approach as compared to a face to face approach:

“*… I don’t think it would really be a long-term answer, but it’s certainly helpful in the interim, yes … it’s really because you can’t actually see someone and you’re not sitting in the same room as someone. There’s nothing beats personal contact and a conversation over the phone is certainly an awful lot better than no conversation at all … but, yeah, a little bit of personal contact would be more advantageous, I think.”(IV5).*

Interviewees commented warmly on the therapists delivering the sessions, describing them as experienced and skilled professionals who were friendly, knowledgeable, empathic and able to quickly establish rapport and put clients at ease. Participants also welcomed the consistency, reliability and convenience of speaking to the same therapist at each session and that times were arranged to suit the needs of the participant. This availability of the therapist was further enhanced by the therapists’ skills in reflecting key details from previous sessions to offer an individualised approach.

For those who had no prior experience of therapeutic support, this down to earth and personalised approach was a welcome contrast to what they had been expecting:

*“She was brilliant. She was just really nice, just really … really nice, really normal. I was a bit concerned it might be a bit airy-fairy and a bit too, you know, psycho-analytic type thing; but no, it wasn’t like that at all.” (IV11).*

All participants confirmed they had received the accompanying self-management CBT manual and about two thirds reported using it. Many positive aspects of the manual were identified relating to the content, structure and purpose. For example, participants used the manual as an aide memoire between sessions, to recall sessional advice and to prompt and motivate their daily goals. Notes could also be made for topics to be discussed with the therapist at the next session which could help participants to articulate their thoughts.

The manual was also important for some in helping to foster the connection between mind and body and principles of CBT and in enhancing their understanding of pain triggers in order to identify solutions. For those who already had a reasonable understanding of the mind-body link and CBT approaches, the manual offered reassurance that they were adopting the correct techniques and helped them feel less isolated in their experience:

*“There was a lot of statements in there which you begin to realise, yes, that’s true, I know about that. What it does is, first of all, it assures you that your circumstances are not unique, and that what you’re experiencing is not the worst thing ever in the world because other people are experiencing it as well … what’s happening to you is happening to other people as well, and that’s rather comforting.” (IV5).*

In suggesting ideas to improve the manual some had found it ‘difficult to navigate’ and others ‘repetitive’ or ‘too rigid’ – which was contrasted with the personalised and adaptable approach of the therapist. Some would have liked more information at the outset about the purpose of the manual alongside the therapeutic sessions and how this would be used by the therapist and client throughout the intervention. Ultimately though, what was important was that the manual was offered alongside the therapeutic support of the therapist:

*“You look at it initially and you think, is this really going to work? But actually for me, what made it work was [therapist]. If you had given me the booklet on my own and had said, right you go away and study that and you give it a go, no it wouldn’t have worked. That would have probably turned me off quite quickly. Having [therapist] there though and being able to speak with him and him explaining how it works and what I needed to do and us then sitting down the following week and discussing things … having his backup and his kind of understanding was without a shadow of a doubt what helped it along.” (IV24).*

### Intervention impact and acceptability

The third and final theme, intervention impact and acceptability, describes post-intervention changes reported by participants and intervention acceptability.

#### Post-intervention changes reported by participants

The majority of participants described positive changes in either their subjective level of pain or their pain-management post-intervention and many attributed the positive change directly to the telephone support intervention. Six participants attributed positive change to something other than the intervention and two felt they had improved due to a combination of the telephone intervention plus something else such as telephone therapy plus acupuncture or medication.

For the participants reporting positive changes directly attributed to the intervention, nine of these reported lower or more manageable levels of pain which seemed to relate to an increase in self-awareness and self-management of symptoms and evidence of cognitive re-structuring.

*“What was interesting is how those sessions got me to think...well, I suppose, basically, sort of, empowered me by showing me how to gain control over what actually what is the reality, what was going on in my head and what I was actually physically experiencing, and I realised that the pain wasn’t that disabling and not that often, it was the thought of it I suppose and the depressing thought of it and, yes, when the pain’s bad it is very bad but then I saw that...I saw a pattern. I started to see things that were causing it.” (IV1).*

*“It’s definitely better now than it was when I started. I still get little moments of it, but it’s not impacting on my daily life at all. Not stopping me doing anything … I think there is a move in attitude for me. I think I’m less anxious and less worried about it than I was … I think having worked through it with everything that research programme and everything that went round it, you know that if you get it back, you know it can be worked through again and go away..” (IV30).*

Participants also valued having someone to share their experience with, without which, the pain could have been an isolating experience:

*“I felt quite good talking about it to be quite frank with you, you know? I’ve got nobody else to speak to about it, and I certainly never tell my family. You know, I keep my pain to myself. Who wants to sit and listen to people moaning, you know? Nobody. But that’s why I really enjoyed talking to [therapist], ‘cause I could explain it all better to her you know, the type of thing I was suffering.” (IV21).*

For the remainder of the participants reporting positive changes directly attributed to the telephone intervention their pain was still present but they had changed the way they thought about their pain and were now able to “put things in perspective”, “think of others worse off” or to focus less on the pain:

*“I’d definitely say it helped. The pain I wouldn’t say went away, but probably I wasn’t as focused on it. I didn’t get as grumpy about it; I didn’t get into a mindset of blaming it for why I couldn’t exercise, why I was feeling low, getting irritable, all these sorts of things. That probably was a help.” (IV14).*

Understanding the link between stress and pain was a key strategy and had resulted in the adoption of more realistic goals and increased self-management. Some participants talked of re-focusing, of understanding their pain more, and feeling more confident to move, being more active and less passive, particularly via the use of goal setting strategies and modifications.

*“I’ve very much learnt … like, I take things in small chunks, and I will rest when I need to rest, and therefore I can do more; and it’s really about managing it and, kind of, understanding myself when I’m starting to struggle; because I’m really bad for pushing through and just trying to keep going.” (IV11).*

For the six participants who attributed their reduced or more manageable levels of pain to something other than the trial intervention the reasons cited included medical (receiving an alternative diagnosis; change in medication; access to new medical treatments); physical (physiotherapy; working less hours; reduction in exercise regime; acupuncture) and psychological (adopting a more positive attitude) interventions outside of the trial treatment.

Four participants who felt there had been no changes post-intervention were still positive about the potential benefits for others from the intervention. In one case the trial was felt to be unsuitable as the participant did not consider their main source of pain to be musculoskeletal although they did have a diagnosis of osteoporosis; in three other cases the intervention was considered to have effected little or no change as the participants were already using cognitive and pain management strategies prior to being recruited into the trial.

Thus, whilst the trial intervention appeared to be less suitable for a small number of participants, it was clear that all found this type of intervention acceptable in principle and all thirty-two participants said they would recommend the intervention to a friend or family member in similar need.

Based on the feedback in this study about wider acceptability some caveats on intervention acceptability and suitability were suggested by participants which might be considered prior to any intervention roll-out – these external factors, and how best to overcome them, as suggested by participants, are summarised in Table 3 below:

**Table 3:** External factors which might influence intervention acceptability and suitability

Factors affecting intervention acceptance	Methods to address these factors
Scepticism and resistance	Clear information at screening about the nature and style of the intervention (cognitive behavioural therapy) and about the link between what we do, the way we think and our physical symptoms
Some will be sceptical and resistant to the idea of a ‘counselling’ approach to preventing chronic widespread pain	
Timing	Intervention impact may be increased if offered earlier rather than later, for example, when participants are experiencing low to moderate pain.
Timing of the intervention offer could impact on acceptability and suitability	
Baseline Knowledge	Intervention screening should include assessment of baseline knowledge and existing use of self-help and CBT pain management techniques
Intervention most useful for those with little or no prior experience of CBT pain management techniques	
Presenting Symptoms	Intervention screening should include assessment of symptoms experienced. Intervention is likely to be most helpful for those with Musculoskeletal pain.
The presenting symptoms experienced may impact on acceptability and suitability	

## Discussion

The present study sought to explore patients’ experiences of tCBT to prevent chronic widespread pain with a view to understanding their potential influences on treatment acceptability. Interviews were undertaken with research trial participants allocated to the intervention arm in a two-arm randomised trial testing a course of tCBT against usual care for CWP.

Our data suggests that a tCBT intervention is acceptable and can have a positive impact based on patients’ subjective qualitative assessments of changes post-intervention. This is particularly important since almost half the sample described their pain as chronic or debilitating prior to receiving the intervention and were accessing a vast range of NHS and alternative provision treatments.

This reported impact is of particular note given the initially sceptical mind-set of about a third of the sample prior to participating in the trial. Whilst many were “desperate for help” and “willing to try anything” some were unsure of the link between mind and pain or doubted that the intervention would have any personal benefits. This finding is consistent with other research exploring acceptability of a CBT intervention for (orofacial) pain which found barriers to engagement could include participants initially considering the intervention to be not appropriate for them, for example due to associations between CBT and depression [24]. In that study, as with the present study the belief that the study could help others could overcome this scepticism, thus displaying perhaps, evidence of unconditional altruism [25]. As the intervention in this study was delivered in the context of a trial the screening process did not include sharing information with potential participants that a treatment aimed at altering thoughts and behaviours might be effective for symptoms. Consistent with other research, participants in this study appeared to have assessed the potential for gain versus risk when considering whether to participate in the study [10].

When asked to reflect back on their experiences of the intervention the majority of the participants were keen to praise the intervention and its perceived impact demonstrating a significant cognitive shift for some. Patients were on the whole satisfied with the intervention modality (telephone), the therapists, and the accompanying manual although a small proportion would have preferred face to face support. The data also suggests that for many there had been positive changes, either a reduction in pain or a revised attitude towards the pain which made it more manageable. Whilst some linked this change to the wider range of interventions received (including medication and physical therapy), two thirds of those interviewed attributed the change directly to the telephone intervention received. Our findings were consistent with earlier acceptability studies which have found positive appraisals of CBT interventions for pain despite some initial scepticism or pre-conceived ideas about CBT being a psychological treatment [10].

A particularly interesting finding to emerge from the interviews was some participants’ interpretation of this being a study about the treatment of existing pain rather than about *preventing* the onset of chronic widespread pain. This was somewhat unexpected given the screening criteria and overall trial aims. It is possible that these participants saw the intervention as managing their CWP which they experience intermittently and indeed this type of intervention has been used to manage CWP. We do not feel that this makes the intervention any less appropriate. In addition, the acceptability study sample did have more pain prior to recruitment, as indicated by the number of pain sites identified in a baseline screening questionnaire – a median of 6 in those interviewed (IQR 3–7.5) compared to 4 in the rest of participants (IQR 3–6). So those interviewed were perhaps less likely to view the study as a preventative trial due to their existing levels of pain.

This study adds to the literature by extending our understanding of the acceptability of interventions for chronic widespread pain in a preventative rather than a treatment setting and adds to the wider research literature on the suitability of psychosocial interventions for patients experiencing pain [10, 24].

### Study limitations

The views expressed in this qualitative study are those of a sample (11%) of the trial cohort who completed at least one treatment session when invitations to the acceptability study were sent and who had not formally withdrawn from treatment or trial without mutual agreement of the therapist (*n* = 302). Those who did formally withdraw (*n* = 172) or had not completed at least one treatment session (*n* = 27) may have found the intervention less acceptable. However, we know from the earlier MUSICIAN study [4, 26] that around 1 in 3 people randomised to studies of the management of chronic widespread pain will not engage with CBT. The current study is a prevention study and so it is not surprising that the engagement is less since a) people might see less relevance in a prevention study and b) the identification of people at high risk will have identified some people who are not at high risk of developing CWP (since high risk is defined on a group basis). So to have 60% of people engaging in such a prevention study is very positive and is in line with the assumptions made prior to the trial getting underway. It could also be argued that any sample in a trial cohort is to an extent skewed since by agreeing to be randomised to a tCBT trial they are likely at the outset to find the intervention somewhat acceptable. In addition, the cross-sectional nature of the study means that data was only collected at one point in time after follow up and further longitudinal studies will be needed to ascertain if the type of gains noted can be sustained in the longer term after this type of intervention.

However, these limitations should not detract from the importance of incorporating nested qualitative studies within trials as they afford participants the opportunity to raise issues not otherwise captured as highlighted in feedback from two participants in this acceptability study about the limitations of the main trial outcome measures in capturing their treatment gains.

A future study might seek to also incorporate the views of the therapists delivering the intervention, particularly given the observation that this was largely viewed as a pain rather than prevention trial. Incorporating the therapists’ views in this study would have allowed a deeper exploration of this interpretation and how it may have impacted on treatment impact and acceptability.

## Conclusions

This study extends our understanding of the acceptability and suitability of preventative interventions for chronic widespread pain and provides information on aspects which may be modified in the future to further enhance treatment acceptability and effectiveness.

Our analysis revealed that participants that completed treatment may have initially either self-managed or adopted a range of other options for pain management prior to starting tCBT. Although they may have thought themselves unsuitable for this type of treatment, or thought that it might be ineffective, they were willing to engage based on the novelty and low-risk. While they may have initially had low expectations for treatment effectiveness, they found their experience was positive, highlighting the treatment modality, qualities of the therapist and the level of contact compared to other experience of health care. Those experiencing a positive effect of the treatment did so through changes in attitude. Steps were identified that could improve acceptability of treatment for participants including assessing prior knowledge of this treatment and giving information about it at screening.

## Additional files

Additional file 1: Participant Flow Diagram. File detailing flow of participants from trial to nested qualitative study. (PDF 280 kb)

Additional file 2: Interview Schedule. (PDF 139 kb)

Additional file 3: Acceptability Study Theoretical Framework. (PDF 404 kb)

## Abbreviations

BABCP: British Association for Behaviour and Cognitive Psychotherapies
CBT: Cognitive Behavioural Therapy
CI: Confidence Interval
CWP: Chronic Widespread Pain
IQR: Inter-Quartile Range
MAmMOTH: Maintaining Musculoskeletal Health
RCT: Randomised Controlled Trial
SD: Standard Deviation
tCBT: Telephone Cognitive Behavioural Therapy
