# Supplementary figures and images for: Telephone cognitive behavioural therapy to prevent the development of chronic widespread pain: a qualitative study of patient perspectives and treatment acceptability
Source: BMC Musculoskelet Disord. 2019 May 10;20:198. doi: 10.1186/s12891-019-2584-2 (PMC6511117; doi:10.1186/s12891-019-2584-2)

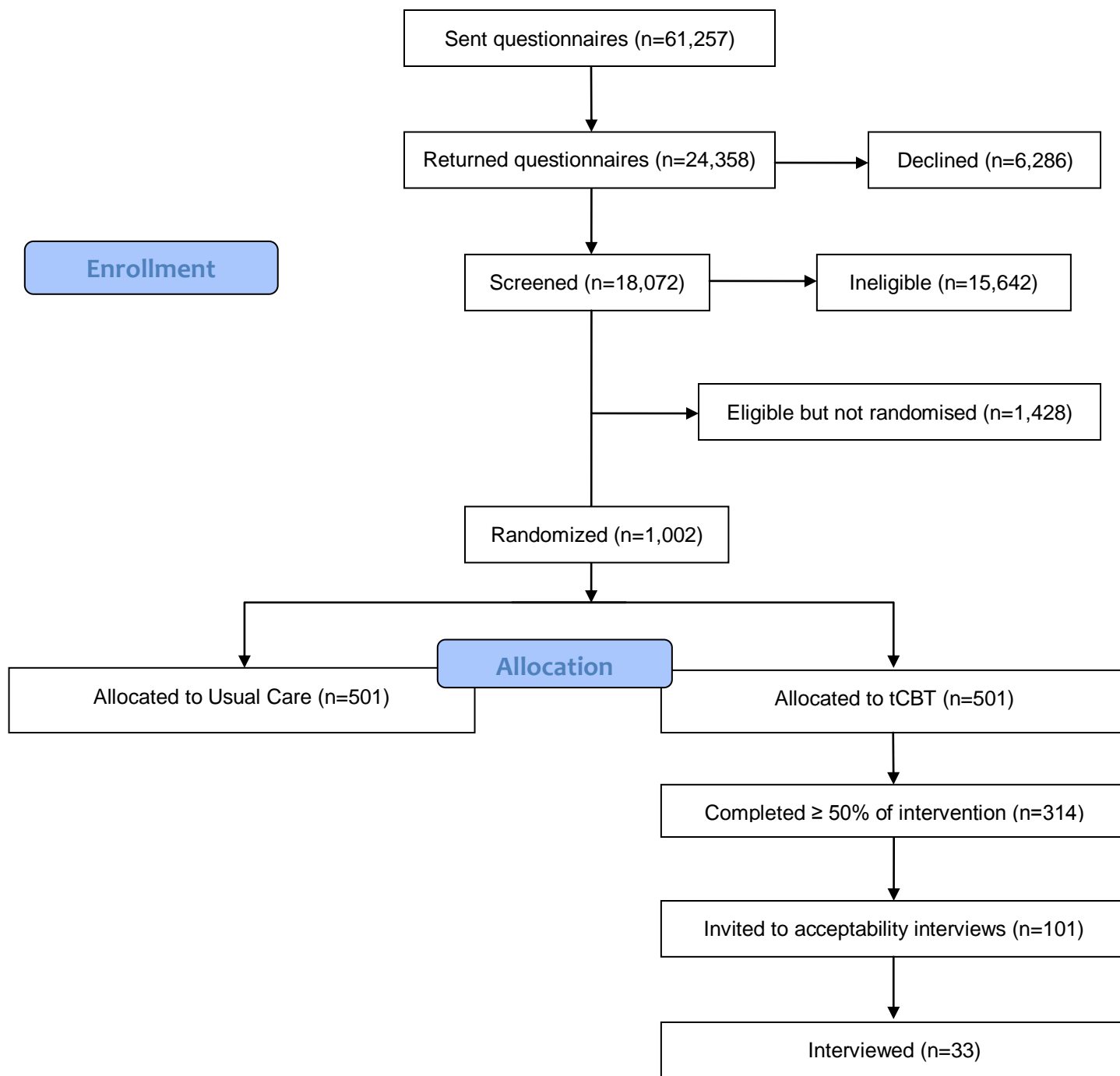

Supplement: Supplementary file 1 — Participant Flow Diagram. File detailing flow of participants from trial to nested qualitative study. (PDF 280 kb) [file 12891_2019_2584_MOESM1_ESM.pdf]
