# Supplementary material for: Telephone cognitive behavioural therapy to prevent the development of chronic widespread pain: a qualitative study of patient perspectives and treatment acceptability
Source: BMC Musculoskelet Disord. 2019 May 10;20:198. doi: 10.1186/s12891-019-2584-2 (PMC6511117; doi:10.1186/s12891-019-2584-2)
Supplement: Supplementary file 2 — Interview Schedule. (PDF 139 kb) [file 12891_2019_2584_MOESM2_ESM.pdf]

MAmMOTH - Acceptability Study

Interview Schedule

Introduction

- Explanation of the MAmMOTH study & objective of acceptability sub study
- Structure and duration of the interview
- Explanation of ethics, consent and confidentiality of interview and analysis
- Any questions?
- Record consent

Context

- Why did you decide to take part in the MAmMOTH study?

  Probe specific benefits to self or to others (i.e., helping the research agenda; to find new ways of working with pain etc)

- If applicable:
  - How have your difficulties affected you day to day?
  - What help have you had in managing your difficulties prior to taking part in MAmMOTH?

  Probe - have they sought prior help (formal or informal) for their pain, what was it, how was it accessed, what was the outcome?

Process (for CBT) telephone

- What were your initial expectations of the telephone therapy service? What did they think it was about?
- What do you now think about accessing this kind of service over the telephone?
- How did your sessions take place and what did you think about the format and frequency of these sessions?

  Probe - How often? How long?

- Who did you speak to?
- (Therapist) Was there anything that you liked or disliked about your therapist?

Probe - specific intervention factors (i.e., goal setting, knowledge, pain management interventions) and non-specific factors (i.e., warmth, empathy, listening etc.)

- Would you have preferred to have met your therapist in a different way?

  Probe - If so, why and what do you think might have been the advantages and disadvantages of meeting in different ways?

- (The manual) Did you receive the manual?
- What did you think of it?

  Probe - usefulness, relevance/helpfulness of stories, choice of interventions, information on CWP, layout, ease of reading

  Probe how often used – i.e., read once or not at all, read throughout, did the book add anything, did they use the diaries and how often?

Outcome

- How are you feeling now, has anything changed since you started the study?

  Probe – if applicable - levels of pain, functioning (social, occupational, private, leisure, family relationships), Mood/depression, anxiety stress

- If applicable (if difficulties identified earlier) - Are you finding it easier or more difficult to manage your everyday life and the way you feel at the moment?
- What do you think might account for this?

  Probe specifically i.e., treatment (and if so, which parts, the book, the therapist?) or other factors such as change in circumstances, other treatments etc,

- Would you recommend this telephone service to a friend/family member who was experiencing similar difficulties?

  Probe – reasons for their response

Ending

- Thank participant for their time and information
- Ask if they would like to receive a copy of the results
