# Supplementary material for: Telephone cognitive behavioural therapy to prevent the development of chronic widespread pain: a qualitative study of patient perspectives and treatment acceptability
Source: BMC Musculoskelet Disord. 2019 May 10;20:198. doi: 10.1186/s12891-019-2584-2 (PMC6511117; doi:10.1186/s12891-019-2584-2)
Supplement: Supplementary file 3 — Acceptability Study Theoretical Framework. (PDF 404 kb) [file 12891_2019_2584_MOESM3_ESM.pdf]

MAmMOTH Acceptability study Theoretical Framework

1.0 Presenting Issues and Pain Management

- 1.1 Nature of the pain experienced
  - 1.1.1 Musculoskeletal
  - 1.1.2 Other health conditions
- 1.2 Pain management strategies
  - 1.2.1 Chronic pain
  - 1.2.2 Discomfort
  - 1.2.3 Minimisation
- 1.3 Help received prior to MAmMOTH
  - 1.3.1 Medical interventions
  - 1.3.2 Physical interventions
  - 1.3.3 Psychological interventions

2.0 Expectations & Reflections

- 2.1 Trial perceptions and motivation to participate
  - 2.1.1 An alternative approach
  - 2.1.2 Altruism
  - 2.1.3 Pain relief
- 2.2 Initial expectations of intervention by phone
  - 2.2.1 Low expectations
  - 2.2.2 Open-minded
  - 2.2.3 High expectations
- 2.3 Post intervention reflections
  - 2.3.1 On treatment modality (phone)
  - 2.3.2 On the therapists
  - 2.3.3 On the manual

3.0 Intervention Impact & Acceptability

- 3.1 Perceived impact: Post intervention changes
  - 3.1.1 Positive change recorded
  - 3.1.2 Impact on daily life
  - 3.1.3 No change recorded
- 3.2 Acceptability: What works
  - 3.2.1 Key elements of intervention success
  - 3.2.2 Acceptability: recommendation to others
  - 3.2.3 Sustaining changes over time
